# Fertility Preservation in Women with Endometriosis

**DOI:** 10.3390/jcm12134331

**Published:** 2023-06-28

**Authors:** Sabrina Rangi, Christine Hur, Elliott Richards, Tommaso Falcone

**Affiliations:** Cleveland Clinic Department of Obstetrics and Gynecology, Women’s Health Institute, 9500 Euclid Ave, Cleveland, OH 44195, USA; rangis2@ccf.org (S.R.); hurc@ccf.org (C.H.); richare@ccf.org (E.R.)

**Keywords:** endometriosis, fertility preservation, infertility

## Abstract

Several mechanisms have been implicated in the pathogenesis of endometriosis-related infertility. For patients considering surgery, the risk of iatrogenic injury is among the most important factors in the context of fertility preservation, along with age and individual reproductive goals. In the case of endometrioma excision, evidence overwhelmingly demonstrates the negative impact of surgery on ovarian reserve, with significant reductions in antimullerian hormone (up to 30% in unilateral versus up to 44% in bilateral endometriomas). The surgical endometriosis patient should be thoroughly counseled regarding fertility preservation and discussion should include tissue, embryo, and oocyte cryopreservation options. For the latter, data support cryopreservation of 10–15 oocytes in women ≤35 years and over 20 for those >35 years for a realistic chance to achieve one or more live births. When performing surgical interventions for endometriosis, reproductive surgeons should employ fertility-conserving surgical methods to reduce the likelihood of postoperative iatrogenic diminished ovarian reserve.

## 1. Introduction

Endometriosis is a chronic inflammatory disease that affects approximately 10% of reproductive-age women, with a prevalence of up to 50% in infertile women [1]. It is characterized by the presence of endometrial glands and stroma outside of the uterus, most commonly involving the pelvis, ovaries, and fallopian tubes [2]. The monthly fecundity rate (MFR) for normal reproductive aged couples is up to 30%, but in infertile couples with endometriosis, the MFR decreases dramatically to 2–10% per month [3]. Endometriosis can negatively impact fertility through multiple mechanisms including anatomic distortion of the reproductive tract, reduction in ovarian reserve, decrease in oocyte and embryo quality, and iatrogenic injury during surgery [3]. 

Due to both the pathologic and iatrogenic causes of infertility in endometriosis, patients with endometriosis should be counseled on reproductive planning and the risks of delayed childbearing. Additionally, fertility preservation options should be considered before treatment is undertaken. While fertility preservation initially gained recognition for its role in preserving fertility in oncology patients at risk for premature ovarian failure secondary to gonadotoxic treatments, it is now recognized as a viable option for women affected by other medical conditions known to compromise ovarian reserve, as well as for those who wish to delay childbearing [4,5]. 

In this article, we review the mechanisms of infertility in endometriosis and strategies for fertility preservation, including oocyte cryopreservation and ovarian tissue cryopreservation. Lastly, we discuss surgical techniques that can be utilized to minimize iatrogenic injury to healthy ovarian tissue. 

## 2. Impact of Endometriosis Surgery on Ovarian Reserve

While several mechanisms have been implicated in the pathogenesis of endometriosis-related infertility, including distorted pelvic anatomy, inflammatory mediated changes, and decreased endometrial receptivity, iatrogenic injury from surgical treatment is one of the most impactful factors when considering fertility preservation [6,7,8,9,10,11,12,13,14,15,16,17,18,19]. 

In mild to moderate endometriosis, randomized control data suggest that surgical treatment may improve live birth and clinical pregnancy rates compared to no treatment. A Cochrane Review from 2014 analyzing three RCTs found that laparoscopic treatment for stage I-II endometriosis was associated with an increase in live birth and clinical pregnancy rates compared to diagnostic laparoscopy [20]. While surgery may be beneficial for fertility in mild cases, the data show it may not be beneficial in endometriosis patients with advanced disease. 

In the case of endometriomas, consideration for fertility preservation is especially prudent in a patient desiring future fertility since evidence overwhelmingly demonstrates the negative impact of surgery, including damage to the ovarian cortex with decrease in ovarian reserve [21,22,23]. These findings are supported by significant reductions in AMH levels following endometrioma excision, especially in women with bilateral endometriomas (up to 30% in unilateral versus up to 44% in bilateral endometriomas) [24,25]. 

While data from several RCTs support endometrioma excision prior to spontaneous conception, the overall evidence does not support endometrioma surgery prior to IVF [26]. Several systematic reviews have shown that women who undergo endometrioma excision have similar IVF outcomes to women with no surgical treatment, including number of oocytes retrieved, live birth rate, and cumulative pregnancy rate [27,28,29]. Given these findings, endometriomas are not routinely excised prior to IVF; however, it is important to note that several of these studies did not take into account endometrioma size and an individualized approach can be taken, particularly in cases where surgery may improve access for oocyte retrieval and prevent spillage of endometrioma contents [6]. It is important to note that currently there are no RCTs evaluating spontaneous conception and IVF outcomes in cases of DIE resection and observational data are mixed [30,31,32]. Overall, expert consensus recommends IVF rather than surgery for women with DIE who desire fertility [33]. However, in women with infertility and pain, shared decision making may prioritize a surgical approach. 

It should be emphasized that repeat surgery does not improve fertility outcomes and IVF should be pursued prior to additional surgery for endometriosis unless pain is also a priority for the patient [34,35]. The surgical endometriosis patient should be thoroughly counseled regarding the risks and benefits of operative management, including its impact on future fertility and the potential for a decline in ovarian reserve. Additionally, the patient can be offered the option for fertility preservation, especially if they are at risk of iatrogenic injury of the ovarian reserve. If surgery is pursued, meticulous effort should be taken to utilize surgical techniques to minimize damage to the ovarian reserve. 

## 3. Unique Challenges Posed by Endometriosis in Fertility Preservation

Fertility preservation can be challenging in endometriosis patients due to baseline diminished ovarian reserve and reduced oocyte/embryo quality. It is well established that ovarian endometriomas can have detrimental effects on ovarian reserve by mechanically stretching the ovarian cortex and causing inflammatory mediated damage [20,21]. These effects are evident on histologic evaluation of affected ovaries, which demonstrate reduced follicular density, increased atresia, and increased primordial follicle activation when compared to an unaffected ovary [36,37]. 

These negative effects of endometriomas on ovarian reserve are reflected by decreased baseline AMH levels in women with endometriomas compared to healthy controls [38]. Additionally, the decline in AMH levels is more pronounced in women with bilateral endometriomas compared to those with unilateral endometriomas [39]. However, the clinical significance of these findings is unclear. AMH is a poor predictor of spontaneous conception and, additionally, women with endometriomas undergoing IVF have similar clinical pregnancy and live birth rates compared to women without endometriomas, despite decreased response to gonadotropins and decreased oocyte yields [27,28]. 

A second challenge posed by endometriosis patients is the potential decreased oocyte and embryo quality. A systematic review published by Sanchez et al. reported that the available evidence demonstrates that endometriosis is associated with a reduction in the number of mature oocytes retrieved compared to other causes of infertility [40,41,42,43,44,45,46]. Additionally, there is a reduction in fertilization rates associated with minimal/mild disease compared to moderate/severe endometriosis [47,48]. Furthermore, embryos derived from patients with endometriosis exhibit disordered development, including mitochondrial dysfunction, decreased mitochondrial mass, slower rates of growth and higher rates of arrested development [14,49,50,51,52]. The clinical significance of these findings is demonstrated by donor oocyte studies, which demonstrate reduced implantation and pregnancy rates in healthy women who undergo transfer with embryos from women with advanced endometriosis compared to healthy controls [53,54,55]. Despite these unique challenges, most studies show that endometriosis has minimal impact on IVF outcomes when compared to women with other causes of infertility [56,57]. These findings highlight the role of IVF, as well as other methods of fertility preservation, in maximizing fertility for endometriosis patients.

## 4. Fertility Preservation in Endometriosis 

Given the negative impact of endometriosis on fertility and the overall favorable IVF outcomes seen in these patients, fertility preservation can be considered in select patients, particularly the surgical patient at risk of diminished ovarian reserve. Techniques include oocyte/embryo cryopreservation and ovarian tissue cryopreservation. Additionally, it is important to consider surgical techniques to decrease iatrogenic injury to the ovarian reserve. 

### 4.1. Oocyte and Embryo Cryopreservation

The evidence supports oocyte cryopreservation as an effective means to preserve fertility in endometriosis patients in young patients, especially when performed prior to surgical management. 

In 2009, Elizur et al. published the first case report of fertility preservation for endometriosis [58]. Authors described the case of a nulliparous 25-year-old female with advanced endometriosis and diminished ovarian reserve secondary to multiple prior extensive surgeries. The patient was able to cryopreserve 21 mature oocytes following three cycles of controlled ovarian stimulation [58]. In 2020, Cobo et al. published a large retrospective cohort study, which included 485 patients with endometriosis [59]. The mean age at vitrification was 35.7 ± 3.7 years and the majority of women had advanced stages of endometriosis (97.7%). The study described several important findings: (1) the number of vitrified oocytes per cycle was higher for the non-surgical patients (6.2 ± 5.8) compared to the unilateral (5.0 ± 4.5) or bilateral (4.5 ± 4.4) surgery groups and (2) nonsurgical patients aged ≤35 years had a higher ovarian response (8.6 ± 6.9 versus 5.1 ± 4.8) and cumulative live birth rate (CLBR) (72.5% versus 52.8%) compared to surgical patients of a similar age [59]. Interestingly, these results were supported in a recent prospective study published by Santulli et al. in 2021, which evaluated prognostic factors related to a high oocyte yield in fertility preservation of endometriosis patients [60]. They found that previous history of surgery for ovarian endometriosis and a woman’s age were the two factors that significantly reduced the number of oocytes retrieved (−1.08; 95% CI −2.02 to −0.15; *p* = 0.024 and −0.21; 95% CI −0.41 to −0.01; *p* = 0.039, respectively) [60]. Overall, the existing evidence provides guidance on the type of endometriosis patient who may benefit from ovarian stimulation and the timing of fertility preservation: the young patient (preferably ≤35) with advanced stages of endometriosis ideally prior to surgical management [59].

Despite the favorable evidence in support of oocyte cryopreservation in the young surgical patient with advanced endometriosis, it is premature to recommend its generalized use in all endometriosis patients [5,61]. Major concerns include the limited evidence in three areas: (1) the efficacy of oocyte cryopreservation in endometriosis patients, (2) the knowledge regarding the quality of vitrified oocytes, and (3) the cost-effectiveness of oocyte cryopreservation [5]. One recent study published by Cobo et al. in 2021 attempts to address these knowledge gaps and offers guidance on the number of vitrified oocytes needed to achieve at least one live birth in endometriosis patients, in addition to the quality of oocytes in this population [62]. The study found that women ≤35 had a CLBR of 95% when approximately 22–24 oocytes were used, whereas women >35 had a CLBR of around 80% using the same number of oocytes, overall highlighting the impact of age on reproductive outcomes. To date, this is the only study that evaluates the number needed to freeze specifically in endometriosis patients. Furthermore, they found that in age-matched controls, there was no difference in CLBR between operated versus non-operated patients, or between endometriosis patients versus patients undergoing elective fertility preservation. Unlike in the previously published Cobo et al. study in 2020, the lack of difference in CLBR in these two separate comparisons highlights that when the same number of vitrified oocytes is used, there is no difference in CLBR. These findings suggest that it is oocyte quantity, rather than quality, that is compromised in endometriosis and that age is the most important variable impacting clinical outcomes [62]. 

**Figure 1:**
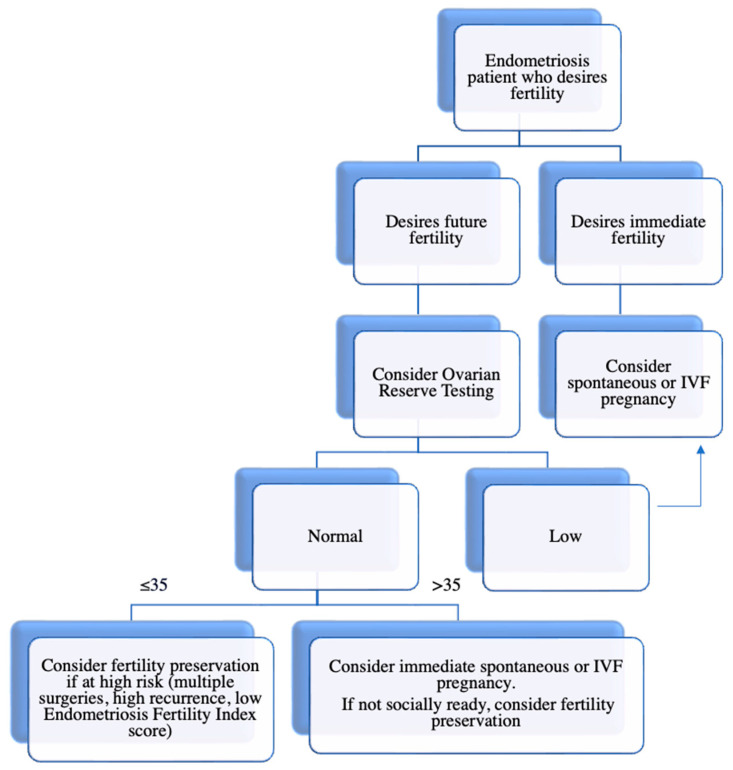
Approach to fertility preservation in an endometriosis patient who desires future fertility.

Despite limited evidence available, the current data provide practical guidance for endometriosis patients who desire future fertility (Figure 1). In summary, a patient with endometriosis with high risk of diminished ovarian reserve should be counseled on oocyte cryopreservation at a young age (≤35), particularly prior to any surgical intervention or if they are at high risk of recurrence [63]. They should be counseled that the two main factors impacting live birth rates are the number of oocytes vitrified and age. It is recommended that women ≤35 year of age should cryopreserve at least 10–15 oocytes to achieve a CLBR between 40 and 70%, which typically can be achieved in 1 or 2 COS cycles [63]. Women over 35 years of age should be counseled to strongly consider immediate spontaneous or IVF pregnancy and, additionally, counseled regarding worse outcomes secondary to age-related fertility decline. Currently, there is limited evidence on the cost-effectiveness of fertility preservation in endometriosis patients and future research in this area, in addition to studies on its efficiency, are needed to better guide care. 

### 4.2. Ovarian Tissue Cryopreservation 

Ovarian tissue cryopreservation (OTC) is a technique for preserving reproductive potential. While previously having been primarily utilized for cancer patients prior to initiation of gonadotoxic therapy, more recently OTC has also been used for other conditions that may adversely impact ovarian function and cause premature ovarian insufficiency (POI). In some cases, OTC may be a reasonable option for patients with endometriosis [64]. In this population, candidates for OTC include those who are unable or choose to forego IVF or in patients who may require an oophorectomy.

There are two techniques of OTC: ovarian cortical tissue cryopreservation and whole ovary cryopreservation. During ovarian cortical tissue cryopreservation, a small volume of cortical tissue containing primordial follicles is removed and then cut into 0.3–2 mm thick pieces and cryopreserved [64,65,66,67,68]. Once restoration of fertility is desired, autotransplantation of the ovarian tissue is performed either in an orthotopic or heterotopic fashion. In orthotopic transplantation, the tissue is attached to the remaining ovary or to the peritoneum of the ovarian fossa. This option may allow for spontaneous conception. In several studies, resumption of normal ovulatory cycles has been reported within 4–9 months [69,70,71,72,73]. A recent review reported 24 live births after orthotopic autotransplantation; however, it is difficult to interpret these results since most women had native ovarian tissue remaining [70]. In heterotopic transplantation, the cortical tissue is implanted in the arm, abdominal wall, or chest wall, and IVF is the only option to achieve pregnancy [74,75]. Successful oocyte retrieval and fertilization with heterotopic autotransplantation has been reported with one live birth; however, no spontaneous pregnancies have been reported [64,76]. Whole-ovary cryopreservation is an option for patients for whom ovarian failure is anticipated [77,78]. Currently, there are no reports of successful transplantation of a previously cryopreserved whole ovary. 

While there are favorable data to support OTC outcomes in women undergoing gonadotoxic treatment, aside from case reports, there is limited evidence of its efficacy in endometriosis patients. The use of OTC in endometriosis was first described in 1999 by Oktay et al. in a patient who underwent orthotopic transplantation of ovarian tissue with subsequent return of ovulation; however, no pregnancy was achieved [79]. Later, in 2005, Donnez et al. described a case of orthotopic OTC in a patient with a 9 cm endometrioma who achieved pregnancy with IVF [80]. Several studies support the use of OTC for indications other than endometriosis, including cancer. In a multicenter retrospective study published by Shapira et al. in 2020, they reported 50 pregnancies (33 spontaneous versus 17 IVF) and 44 deliveries among 60 patients undergoing 70 auto-transplantations [81]. Overall, 50% of women were able to achieve at least 1 pregnancy with 41.6% attaining a delivery. In their cohort, they observed younger women were among those who became pregnant (31.6 ± 5.2 vs. 34.8 ± 6.2; *p* = 0.03) [81]. Despite the promising outcomes of OTC used for other indications, further research regarding its efficacy, risks, benefits, and cost-effectiveness in endometriosis patients is needed prior to more widespread use.

There are several advantages of OTC including the ability to perform it any time in the menstrual cycle and without ovarian stimulation. However, OTC requires two surgical procedures, the first to harvest the tissue, followed by auto-transplantation [82]. Access to OTC may be more limited compared to oocyte cryopreservation since the latter is more routine. Additionally, the quality of oocytes may be impacted when obtained from ovarian tissue cryopreserved from an ovary involving an endometrioma, but more data are needed to address this concern. 

### 4.3. Reproductive Counseling

Counseling and shared decision making is an integral part of endometriosis management and fertility preservation, especially at early stages of the disease and prior to surgical intervention [61]. The provider should elucidate the patient’s goals for endometriosis treatment, in addition to current and future fertility goals, including the number of desired children. Patients should be well-informed regarding the limited evidence to recommend routine fertility preservation in all endometriosis patients; however, if the patient is young and at high risk for diminished ovarian reserve, there is promising evidence to recommend its use. Patients should be counseled regarding the risks associated with IVF and/or surgery and the higher potential for procedural risks in patients with advanced disease secondary to distorted anatomy, pelvic adhesions, and large ovarian cysts [61]. It is important to counsel patients that oocyte cryopreservation does not guarantee pregnancy and that multiple COS cycles may be required to optimize egg banking. Given the lack of universal coverage of oocyte cryopreservation in the United States and the limited evidence evaluating its cost-effectiveness in endometriosis patients, patients need to be counseled on the financial aspects of fertility preservation as well. Aside from the cost, the psychological and physical impacts of repeated cycles, surgery, and endometriosis-related pain should be addressed and provide the basis of an ongoing discussion with the patient.

### 4.4. Alternative and Complementary Strategies 

When considering fertility preservation for endometriosis patients, it is useful to consider alternative approaches to manage endometriosis-related infertility, including medical treatment, intrauterine insemination, third-party reproduction, and adoption. 

While medical treatment is effective in reducing pain in endometriosis patients, it has a very limited role in endometriosis-related infertility and does not improve fertility outcomes. In three out of four studies described in a 2014 Cochrane Review, there was no evidence of improved IVF outcomes and clinical pregnancy rates between women who received GnRH agonists versus antagonists, ovulation suppression versus placebo, and pre-surgical medical therapy versus surgery alone [28,83,84,85]. Only one study found a significant increase in clinical pregnancies among women who received 3 months of GnRH agonist pretreatment prior to IVF compared to the those who did not; however, this evidence was noted to be of very low quality [86]. While this latter study did show a beneficial role of GnRH pretreatment, further evidence is needed and its use must be weighed against the risk of additional costs, possible side-effects, and the potential to delay pregnancy [6]. 

Aside from medical treatment, intrauterine insemination (IUI) may represent an alternative option for patients with endometriosis-related infertility, especially in those who may not be able or desire to undergo IVF. Additionally, IUI has several advantages, including its simplicity and lower associated costs compared to IVF. Early studies investigating IUI outcomes in women with endometriosis versus unexplained infertility demonstrate conflicting results, with several studies reporting lower pregnancy rates in endometriosis patients [87]. However, many of these studies have been noted to have significant methodological weaknesses and their findings must be considered with caution. More recently, a retrospective study published in 2022 found that in 494 IUI treatment cycles with ovarian stimulation, there were no significant differences in clinical pregnancy and live birth rate per cycle between the minimal/mild endometriosis and unexplained infertility groups [88]. Additionally, a subgroup analysis found no significant difference in the live birth rate in women who underwent surgical intervention versus no intervention. In summary, their findings suggest that there is no impact of early endometriosis on IUI with ovarian stimulation, highlighting the potential role of IUI for select patients [88]. While further rigorous prospective data are needed, especially taking into consideration advanced stages of endometriosis, there is current evidence to support the role of IUI in endometriosis-related infertility. 

Lastly, in certain circumstances, third-party reproduction and adoption may be alternative strategies for select individuals with endometriosis-related infertility. For third-party reproduction, benefits include involvement in the reproductive process and the possibility of using one’s own genetic material, specifically in surrogacy. While there is currently no data of its use specifically in endometriosis patients, these options should be reviewed and offered to appropriate patients. 

## 5. Surgical Techniques to Minimize Iatrogenic Effects on Ovarian Reserve

In the surgical endometriosis patient desiring future fertility, care should be taken to minimize the iatrogenic effects of surgery on ovarian reserve. Several surgical techniques can be utilized to minimize ovarian injury, including minimization of electrosurgery and avoiding repeat surgery.

### 5.1. Cystectomy Versus Ablation

In the case of endometriomas, randomized control data demonstrate that cystectomy results in improved rates of pain resolution, reduced recurrence rates, and improved spontaneous conception compared to ablative approaches [26]. Currently, cystectomy is the standard surgical approach for endometriomas. Cystectomy first involves identifying the plane between the endometrioma and the ovarian cortex to minimize injury to the healthy cortex. This plane can be difficult to identify secondary to fibrosis and inflammation, so one can consider using dilute vasopressin to hydrodissect and separate the cyst wall from the ovarian stroma. Controlled traction and countertraction should be used to peel the endometrioma from the cortex in order to avoid forceful tissue separation. If bleeding at the ovarian hilum is encountered, electrosurgery should be used sparingly. Several studies demonstrate a benefit of suture or hemostatic sealants over electrosurgery to minimize injury to the ovarian reserve [89,90,91,92]. 

Despite several improved outcomes, including rates of pain resolution, spontaneous conception, and recurrence rates, a cystectomy results in greater injury to the ovarian reserve than ablative approaches. In women who are at risk of diminished ovarian reserve, ablation may be a favorable approach compared to cystectomy. Ablation can be achieved through different sources including monopolar, bipolar, plasma, and CO_2_ laser. Overall, plasma energy and CO_2_ laser results in less injury to the ovary than monopolar or bipolar electrosurgery [93,94]. 

Several studies have investigated whether combined cystectomy and ablation techniques reduce ovarian injury when compared to either method alone. Donnez et al. described a three-step approach: (1) laparoscopic biopsy and cyst drainage to confirm endometriosis diagnosis; (2) 12 weeks of a gonadotropin receptor hormone (GnRH) agonist to decrease the size of the endometrioma; and (3) laparoscopic ablation withCO_2_ laser of the remaining cyst wall [95]. A small randomized controlled trial (RCT) demonstrated a smaller post-operative decline in AMH in the three-step approach compared to traditional cystectomy alone [96]. Despite these findings, pregnancy outcomes were not evaluated and the need for multiple surgeries limits the utility of a three-step approach. 

In 2010, a subsequent combined technique was described by Donnez et al. with only two steps: (1) cystectomy of 80–90% of the endometrioma, followed by (2) laser vaporization of the remaining cyst wall [97]. A prospective cohort study evaluated the ovarian volume and antral follicle count (AFC) between the operated and non-operated ovary 6 months after surgery and found no significant differences, suggesting that a combined approach may improve preservation of the ovarian reserve [97]. Despite this, an RCT compared the two-step approach to cystectomy alone and failed to find any differences in the AFC between the two groups at 1, 3 and 6 months after surgery [98]. Larger RCTs are needed to evaluate a combined approach and its impact on ovarian reserve.

### 5.2. Techniques for Hemostasis 

Bipolar electrosurgery is commonly used to control bleeding at the ovarian hilum following cystectomy; however, bipolar energy can cause iatrogenic injury to adjacent ovarian follicles in healthy cortical tissue. Randomized trial data demonstrate a benefit of alternative hemostatic methods, including suture and hemostatic sealants, compared to electrosurgery, to reduce iatrogenic injury to the ovarian reserve.

In 2016, a RCT compared bipolar electrosurgery to suture in women undergoing unilateral endometrioma surgery and found that AMH levels were significantly higher in the suture group compared to the bipolar group 3 months after surgery [90]. A second RCT demonstrated similar results for bilateral endometriomas with a larger decline in AMH in the bipolar group compared to the suture group (however, this difference did not meet statistical significance) [99]. These two studies demonstrated a decline in AMH after endometrioma surgery in both surgical groups; however, the data favor the use of suture to preserve healthy ovarian tissue. 

Topical hemostatic agents can also be used to control bleeding. Overwhelmingly high-quality data demonstrate a significantly smaller postoperative decline in AMH when hemostatic sealants are used compared to bipolar electrosurgery [91,92,100]. A meta-analysis from 2015 pooled several RCTs comparing suture, hemostatic sealants, and bipolar surgery and found that suture and hemostatic sealants preserved ovarian reserve over electrosurgery [89]. Taken together, bipolar coagulation should be used sparingly and alternative hemostatic methods should be employed in order to decrease the negative effects of surgery on ovarian reserve. 

### 5.3. Anti-Adhesion Barriers

An important consideration at the time of surgery is the formation of postoperative pelvic adhesions. As discussed previously, adhesions have the potential to distort reproductive anatomy and contribute to infertility and pain. While adhesion barriers are not FDA approved in the setting of laparoscopic surgery, their use has been described in the literature and several studies offer a reasonable approach to their use in endometriosis surgery. In a 2015 Cochrane Review of 18 RCTs, oxidized regenerated cellulose (Interceed), expanded polytetrafluoroethylene (Gore-Tex), and sodium hyaluronate with carboxymethylcellulose (Seprafilm) were associated with a reduction in adhesion formation [101]. In a 2020 Cochrane update, Ahmad et al. found that none of these studies reported the efficacy of their use in improving clinical outcomes such as pelvic pain or live birth rate [102]. Of the studies that demonstrated a reduced risk of adhesion formation, all were deemed to be of low evidence. In summary, the research suggests that there is no conclusive evidence of the effectiveness of adhesion barrier use; however, since no adverse events have been associated with their use, it may be reasonable to use anti-adhesion barriers at the time of endometriosis surgery to reduce the formation of postoperative adhesions. Overall, further rigorous data are needed to evaluate their efficacy. 

### 5.4. Surgeon Experience

When performing endometrioma surgery, data suggest that the expertise of the surgeon is significant. A multicenter, prospective trial evaluated endometrioma cyst wall specimens after laparoscopic removal and found that less experienced surgeons inadvertently removed more healthy ovarian tissue than more experienced surgeons [103]. A second retrospective study from Taiwan demonstrated improved live birth rates after cystectomy when it was performed by an experienced surgeon compared to trainee [104]. Neither of these studies evaluated the impact on ovarian reserve as measured by AMH levels; however, the data demonstrate that surgeon proficiency plays a role in minimizing the loss of healthy ovarian tissue. Endometrioma surgery requires meticulous technique, and an experienced surgeon may be more adept at preserving ovarian architecture. 

## 6. Areas of Future Study

While this review highlights what is known regarding fertility preservation in endometriosis patients and the various surgical techniques that can be employed, there remain many unanswered questions and challenges. Several potential areas of further research were discussed throughout this review; however, there are a few additional considerations that warrant discussion. For example, little is known regarding the utilization of fertility preservation in endometriosis patients. Further research is needed to determine how often endometriosis patients are counseled and offered fertility preservation and, subsequently, decide to pursue one of the mentioned techniques. Qualitative research on patient satisfaction in those who do pursue fertility preservation is an additional area of interest. Additionally, a cost-effective analysis of fertility preservation in endometriosis patients would offer further insight into what type of patient would benefit from fertility preservation and aid in provider counseling and shared decision making. Lastly, while Cobo et al. made great progress in our understanding of the effectiveness of oocyte cryopreservation in endometriosis patients, further rigorous prospective research is needed.

## 7. Conclusions

There are several approaches for fertility preservation for women with endometriosis. These primarily include oocyte, embryo, and tissue cryopreservation. In addition to offering fertility preservation, it is critical that reproductive surgeons use fertility-preserving surgical techniques to minimize the risk of ovarian reserve damage and improve fertility outcomes. It is important to note that not all women with endometriosis will need fertility preservation techniques, as each patient’s situation is unique; care should be individualized. Overall, fertility preservation techniques can be a valuable option for women with endometriosis who desire future fertility. It is important for gynecologists and reproductive surgeons to be knowledgeable about these options and to discuss them with their patients affected by endometriosis who are considering future fertility.

## Data Availability

Not applicable.
